# Cadmium Accumulation Involves Synthesis of Glutathione and Phytochelatins, and Activation of CDPK, CaMK, CBLPK, and MAPK Signaling Pathways in *Ulva compressa*

**DOI:** 10.3389/fpls.2021.669096

**Published:** 2021-06-21

**Authors:** Alberto González, Daniel Laporte, Alejandra Moenne

**Affiliations:** ^1^Laboratory of Marine Biotechnology, Faculty of Chemistry and Biology, University of Santiago, Santiago, Chile; ^2^Laboratorio Multidisciplinario, Instituto de Ciencias Biomédicas, Universidad Autónoma de Chile, Talca, Chile

**Keywords:** ascorbate, antioxidant enzymes, cadmium, glutathione, marine alga, metallothioneins, phytochelatins

## Abstract

In order to analyze the effect of cadmium in *Ulva compressa* (Chlorophyta), the alga was cultivated with 10, 25, and 50 μM of cadmium for 7 days, and the level of intracellular cadmium was determined. Intracellular cadmium showed an increase on day 1, no change until day 5, and an increase on day 7. Then, the alga was cultivated with 10 μM for 7 days, and the level of hydrogen peroxide, superoxide anions, and lipoperoxides; activities of antioxidant enzymes ascorbate peroxidase (AP), dehydroascorbate reductase (DHAR), and glutathione reductase (GR); the level of glutathione (GSH) and ascorbate (ASC); and the level of phytochelatins (PCs) and transcripts encoding metallothioneins (UcMTs) levels were determined. The level of hydrogen peroxide increased at 2 and 12 h, superoxide anions on day 1, and lipoperoxides on days 3 to 5. The activities of AP and GR were increased, but not the DHAR activity. The level of GSH increased on day 1, decreased on day 3, and increased again on day 5, whereas ASC slightly increased on days 3 and 7, and activities of enzymes involved in GSH and ASC synthesis were increased on days 3 to 7. The level of PC2 and PC4 decreased on day 3 but increased again on day 5. The level of transcripts encoding UcMT1 and UcMT2 increased on days 3 to 5, mainly that of UcMT2. Thus, cadmium accumulation induced an oxidative stress condition that was mitigated by the activation of antioxidant enzymes and synthesis of GSH and ASC. Then, the alga cultivated with inhibitors of calcium-dependent protein kinases (CDPKs), calmodulin-dependent protein kinases (CaMKs), calcineurin B-like protein kinases (CBLPKs), and MAPKs and 10 μM of cadmium for 5 days showed a decrease in intracellular cadmium and in the level of GSH and PCs, with the four inhibitors, and in the level of transcripts encoding UcMTs, with two inhibitors. Thus, CDPKs, CaMK, CBLPKS, and MAPKs are involved in cadmium accumulation and GSH and PC synthesis, and GSH and PCs and/or UcMTs may participate in cadmium accumulation.

## Introduction

Cadmium is a nonessential heavy metal that it is not required for the function of any protein or enzyme in plants and animals, with the exception of carbonic anhydrase of marine microalgae that uses cadmium as a cofactor (Xu et al., 2008; Gallego et al., 2012). Cadmium ions accumulate in the soil and water and are produced by metallurgic industries, urban traffic, cement industries, and waste incinerators (Gallego et al., 2012). Cadmium is magnified in the food chain and is highly toxic for humans causing kidney and liver diseases, and gastrointestinal and lung cancer (Gallego et al., 2012). Cadmium has hydrated ionic radium of 0.97 Å that is similar to hydrated calcium radium of 0.99 Å (Nithingale, 1959). Thus, cadmium is transported through calcium channels and its uptake into cells uses iron, copper, zinc, and magnesium transporters in animals and plants (Gallego et al., 2012).

In plants, cadmium is absorbed by roots through the iron transporters IRT1 and IRT2, and the zinc/iron transporters ZIP2 and NRAMP1 (Vert et al., 2001, 2002, 2009; He et al., 2017). Cadmium is then loaded to the xylem and transported to the leaves by activation of ATP-dependent transporters HMA2 and HMA4 (Verret et al., 2004; Wong and Cobbett, 2009; Sato-Nagasawa et al., 2012; Takahashi et al., 2012). In this sense, it was recently shown that cadmium increased the expression of IRT1, IRT2, HMA2, and HMA4 in *Brassica chinensis* inducing cadmium accumulation in roots (Huang et al., 2021). Cadmium accumulation in plants involve the transporters HMA1, that transport cadmium with GSH to the vacuole (Mendoza-Cózatl et al., 2011), HMA3 that transport free cadmium ions to the vacuole (Sasaki et al., 2014), and ABCC1 and ABCC2 that transport cadmium with phytochelatins (PCs) to the vacuole (Mendoza-Cózatl et al., 2011; Park et al., 2012).

In plants, cadmium induces oxidative stress-activating antioxidant enzymes such as superoxide dismutase (SOD), catalase (CAT), ascorbate peroxidase (AP), and glutathione reductase (GR), and the synthesis of the antioxidant compound GSH (Cuypers et al., 2011, 2016; Romero-Puertas et al., 2019). Cadmium accumulation involved the synthesis of GSH and PCs that are formed by condensed units of GSH, and they are synthesized by the enzyme phytochelatin synthase (PCS) (Yadav, 2010; Brunetti et al., 2011; Yamazaki et al., 2018). Cadmium ions are coordinated by sulfhydryl groups of GSH and PCs (Zhu et al., 1999; Shukla et al., 2012, 2013). In addition, cadmium can be sequestered by small (around 10 kDa) cysteine-rich proteins named metallothioneins (MTs) that coordinate divalent metal ions via sulfhydryl groups of cysteines (Cobbett and Goldsbrough, 2002). It has been shown that plant MTs are involved in the tolerance and accumulation of cadmium and other heavy metals in plants (Zimeri et al., 2005; Pagani et al., 2012; Rono et al., 2021). In addition, oxidative stress induced by cadmium activates MAPK signal transduction pathway that involves MAPKKK that phosphorylates MAPKK that, in turn, phosphorylates MAPK that activate transcription factors and gene expression (Gallego et al., 2012). In maize, cadmium activates ZmMPK3 and ZmMPK6-1; in rice, it activates OsMPK3 and OsMPK6, and several distinct MAPKs in alfalfa (Jonak et al., 2004; Yeh et al., 2007; Liu et al., 2010; Zhao et al., 2013).

In microalgae, four IRT (ZIP) transporters are encoded in the genome of the green microalga *Chlamydomonas reinhardtii* and four in the genome of the red microalga *Cyanidoschizon merolae*, whereas 12 are encoded in *Arabidopsis thaliana* genome (Hainikenne et al., 2005). In addition, HMA1, HMA2, and HMA3 transporters are encoded in *C. reinhardtii* genome, and only HMA1 and HMA2 are encoded in *C. merolae* genome, whereas eight are encoded in *A. thaliana* (Hainikenne et al., 2005). Thus, similar transporters of iron, zinc, and cadmium exist in plants, microalgae, and euglenoids, and may have similar functions. In fact, *Euglena gracilis* cultivated with cadmium, mercury, and lead accumulated these metals, mainly cadmium, and overexpressed transporters such as HMT2 that extrudes metals from the cells and HMT3 involved in the accumulation of these metals in the chloroplasts (Kathiwada et al., 2020). In addition, cadmium induced oxidative stress leading to the increased expression and enhanced activities of antioxidant enzymes such as SOD, CAT, AP, GR, and glutathione peroxidase (GP) in the microalgae *Nannochloropsis oculata*, *Nitzschia palea*, and *Scenedesmus quadricauda* (Lee and Shin, 2003; Figueira et al., 2014; Kovacik and Dresler, 2018). Cadmium accumulation involved the synthesis of GSH and PCs in the diatom *Thalassiosira weissflogii* during the first 6 h of culture indicating that these compounds are the first line of defense against cadmium (Wu et al., 2016). On the other hand, microalgal genomes contain one to several copies of potential MTs gene, but the increase in expression of these proteins in response to heavy metals has been poorly studied (Balzano et al., 2020). Cadmium accumulates in the chloroplasts of *E. gracilis* and involved the uptake of free cadmium, and cadmium/PC complexes to the chloroplasts (Mendoza-Cózatl et al., 2002; Mendoza-Cózatl and Moreno-Sánchez, 2005). Interestingly, *E. gracilis* cells exposed to cadmium overexpressed an MAPKKK, several chaperones, and enzymes that synthesize GSH and PCs, but not expression of an MT (Kathiwada et al., 2020).

In marine macroalgae, it has been shown that the green macroalga *Ulva lactuca* cultivated with increasing concentrations of cadmium ranging from 100 to 700 μM for 4 days showed an inhibition of growth rate, mainly at concentrations higher than 400 μM, and the amount of intracellular cadmium increased linearly with increasing concentrations of cadmium (Kumar et al., 2010). In addition, the level of hydrogen peroxide and lipoperoxides was enhanced with increasing concentrations of cadmium, and the content of chlorophylls was decreased (Kumar et al., 2010). The activities of antioxidant enzymes SOD, AP, GR, and GP were enhanced as the antioxidant molecules GSH and ASC (Kumar et al., 2010). In addition, cadmium also increased the activity of lipoxygenase, the level of polyunsaturated fatty acids C18:3 and C18:2, and the level of the membrane protective polyamine putrescine (Kumar et al., 2010). The green macroalgae *Ulva prolifera* and *Ulva linza* exposed to increasing concentrations of cadmium ranging from 5 to 120 μM showed a decrease in growth rate and in photosynthetic efficiency and an increase in cadmium accumulation (Jiang et al., 2013). *U. prolifera* accumulated more cadmium than *U. linza*, but the latter better tolerates cadmium toxicity (Jiang et al., 2013). The green macroalga *Cladophora glomerata* exposed to increasing concentrations of cadmium for 7 days showed a decrease in growth rate, in chlorophyll content, and in total proteins (Celekli and Bulut, 2020). Moreover, *C. glomerata* showed an increase in lipoperoxides and in the level of the osmoprotectant amino acid, proline (Celekli and Bulut, 2020).

The green macroalga *Ulva compressa*, cultivated with 10 μM of copper, showed an increase in hydrogen peroxide, superoxide anions, and lipoperoxides (González et al., 2010), an increase in GSH level, but a decrease in ASC content (Mellado et al., 2012) and an increase in activities of antioxidant enzymes SOD, AP, and GR (González et al., 2010, 2012). The alga exposed to increasing concentration of copper ranging from 2.5 to 10 μM for 12 days showed a linear increase in intracellular copper and an increase in the level of PCs (Navarrete et al., 2019). In addition, the expression of UcMT1, UcMT2, and UcMT3 also increased in response to copper stress (Navarrete et al., 2019; Moenne et al., 2020; Zúñiga et al., 2020).

In this work, we analyze the effects of increasing concentrations of cadmium in the marine alga *U. compressa* and compare these effects with those induced by copper excess. To this end, the alga was cultivated with 10–50 μM of cadmium for 7 days, and the level of intracellular cadmium was determined. In addition, the level of hydrogen peroxide, superoxide anions, and lipoperoxides, the activities of antioxidant enzymes AP, DHAR, and GR, and the level of PCs and transcripts encoding UcMT1, UcMT2, and UcMT3 were determined in the alga cultivated with 10 μM of cadmium for 0–7 days. Furthermore, the involvement of calcium-dependent protein kinases (CDPKs), calmodulin-dependent protein kinases (CaMKs), calcineurin B-like protein kinases (CBLPKs), and MAPKs in the accumulation of intracellular cadmium, synthesis of GSH and PCs, and in the level of UcMT transcripts were analyzed in the alga cultivated with 10 μM of cadmium for 5 days.

## Materials and Methods

### Algal and Seawater Sampling

The alga *U. compressa* was collected during spring 2020 in Cachagua, a nonpolluted site of central Chile, and transported to the laboratory in a cooler with ice. The alga was washed with seawater, cleaned manually, and sonicated twice for 2 min using an ultrasound bath (Hilab Innovation Systems). Seawater was obtained from Quintay, a nonpolluted site of Central Chile, filtered, and kept at 4°C, in darkness.

### *In vitro* Cultures and Treatments With Cadmium and Inhibitors

For the detection of intracellular cadmium, the alga (5 g) was cultivated in 30 ml of seawater containing 10, 25, or 50 μM of cadmium for 0, 1, 3, 5, and 7 days, in triplicates. Cultures were performed under irradiance of 50 μmol m^–2^ s^–1^ and using a photoperiod of 14-h light:10-h dark, at 14°C. The culture medium was changed every 48 h. Samples (5 g in triplicate) were harvested on days 0, 1, 3, 5, and 7, and sampling occurred 11 h after the start of the day. Samples were washed twice with 50 mM Tris–HCl (pH 7)–10 mM EDTA for 20 min in order to remove copper ions from algal cell walls (Hassler et al., 2004). Samples were dried in an oven at 60°C for 4 days until reaching a constant dry weight (around 2.5 g) and kept at room temperature.

For the detection of GSH, ASC, and lipoperoxides, the alga (5 g) was cultivated in 30 ml of seawater containing 10 μM of cadmium for 0, 1, 3, 5, and 7 days, in triplicate, and the culture medium was changed every 48 h. Samples (10 g in triplicate) were harvested on days 0, 1, 3, 5, and 7 and washed twice with 50 mM Tris–HCl (pH = 7)–10 mM EDTA for 20 min. Samples were dried in an oven at 60°C for 4 days until reaching a constant dry weight (around 2.5 g) and kept at room temperature.

For detection of antioxidant enzyme activities and transcripts encoding UcMTs, the alga (10 g) was cultivated in 60 ml of seawater containing 10 μM of cadmium for 0, 1, 3, 5, and 7 days, in triplicates, and the culture medium was changed every 48 h. Samples (10 g in triplicate) were harvest on days 0, 1, 3, 5, and 7, washed twice with 50 mM Tris–HCl (pH = 7)–10 mM EDTA for 20 min and kept at −80°C.

For detection of intracellular cadmium in samples cultivated with inhibitors and cadmium, the alga (5 g) was cultivated in 30 ml of seawater, 5 μM staurosporine (ST), an inhibitor of CDPKs; KN62 (KN), an inhibitor of CaMKs; FK506 (FK), an inhibitor of CBLPKs, or PD98059 (PD), an inhibitor of MAPKs, and with 10 μM cadmium, for 5 days, in triplicates. The culture medium containing inhibitors and cadmium was changed every 48 h. Samples were harvested on day 5, and sampling occurred 11 h after the start of the day. Samples were washed twice with 50 mM Tris–HCl (pH = 7)–10 mM EDTA for 20 min, and dried in an oven at 60°C for 4 days until reaching a constant dry weight. The detection of GSH and PCs was performed in these samples.

For the detection of transcripts encoding MTs in samples cultivated with inhibitors and cadmium, the alga (5 g) was cultivated in 30 ml of seawater 5 μM ST, KN, and PD, and with 10 μM cadmium for 5 days, in triplicates. The culture medium containing inhibitors and cadmium was changed every 48 h. Samples (5 g in triplicate) were harvested on day 5, washed twice with 50 mM Tris–HCl (pH = 7)–10 mM EDTA for 20 min and kept at −80°C.

### Detection of Intracellular Cadmium

Intracellular cadmium was detected as described in Navarrete et al. (2019). The alga [0.5 g of dry tissue (DT)] was pre-incubated in 6 ml of nitric acid 65% (Merck, ultrapure) and 2 ml of 30% hydrogen peroxide (Merck, ultrapure) in Teflon vials, overnight. Algae were digested in a microwave oven at 200°C for 10 min with 1,800 W of power and at 200°C and for 20 min with 1,800 W of power. Digests were cooled at room temperature for 30 min and diluted with 25 ml of ultrapure water. Samples were filtered with 0.2-μm membranes and analyzed by inductively coupled plasma optical emission spectrometry (ICP-OES, Perkin Elmer).

### Detection of Hydrogen Peroxide, Superoxide Anions, and Lipoperoxides

Detection of hydrogen peroxide was performed as described in González et al. (2010). The alga [1.5 g of fresh tissue (FT)] was incubated in 100 ml of 100 mM Tris–HCl (pH = 7) containing 10 μM of 2′,7′ dichlorodihydrofluorescein diacetate (DCHF) (Calbiochem, San Diego, CA, United States) for 45 min at room temperature in darkness. The alga was rinsed with seawater and dried with paper, weighted, and frozen in liquid nitrogen. The alga was homogenized in liquid nitrogen in a mortar with a pestle. Five milliliters of 100 mM Tris–HCl (pH = 7) was added, and the homogenization was pursued until thawing. The mixture was centrifuged at 10,000 × *g* for 5 min at 4°C, and the supernatant was recovered. Fluorescence of the supernatant was determined using an excitation wavelength of 488 nm and an emission wavelength of 525 nm, and a spectrofluorometer model LS-5 (PerkinElmer, Shelton, CT, United States). Hydrogen peroxide level was expressed as nanomoles of 2′,7′ dichlorofluorescein (DCF), and the calibration curve was prepared with 1 to 500 nmol of DCF.

Detection of superoxide anions was performed as described in González et al. (2010). The alga (1.5 g of FT) was incubated in 100 ml of 100 mM Tris–HCl (pH = 7) containing 100 μM of hydroethidine (HE), for 45 min at room temperature, in darkness. The alga was rinsed with seawater, dried with paper, weighted, and frozen in liquid nitrogen. The alga was homogenized in liquid nitrogen in a mortar with a pestle, 5 ml of 100 mM Tris–HCl (pH = 7) was added, and the homogenization was pursued until thawing. The mixture was centrifuged at 10,000 × *g* for 5 min at 4°C, and the supernatant was recovered. Fluorescence of the supernatant was determined using an excitation wavelength of 480 nm, an emission wavelength of 590 nm, and a spectrofluorometer model LS-5 (PerkinElmer, Shelton, CT, United States). Superoxide anions level was expressed as nanomoles of 2-hydroxyethidium (2-OHE), and the calibration curve was prepared using the extinction coefficient of 2-OHE (ε = 9.4 mM^–1^ cm^–1^).

Detection of lipoperoxides was performed as described in Ratkevicius et al. (2003). The alga (1.5 g of DT) was frozen in liquid nitrogen and homogenized in a mortar with a pestle. Five milliliters of 0.1% (w/v) trichloroacetic acid (TCA) was added, and the homogenization was pursued until thawing. The mixture was centrifuged at 10,000 × *g* for 20 min at 4°C, and the supernatant was recovered. Lipoperoxides were determined by the addition of 200 μl of the supernatant to 800 μl of a reaction mixture containing 0.5% thiobarbituric acid (solubilized in 20% TCA) and an incubation at 100°C for 30 min. The absorbance of the solution was analyzed at 512 nm. The amount of lipoperoxides was determined using the extinction coefficient of adduct formed by malondialdehyde and thiobarbituric acid (ε = 155 mM^–1^ cm^–1^).

### Preparation of Protein Extracts

Protein extracts were prepared as described in Laporte et al. (2020b). The alga (2 g of FT) was frozen in liquid nitrogen and homogenized in a mortar with a pestle. Six milliliters of 100 mM of phosphate buffer (pH = 7) supplemented with 5 mM β-mercaptoethanol was added, and the homogenization was pursued until thawing. The homogenate was centrifuged at 10,000 × *g* for 15 min at 4°C, and the supernatant was recovered. Proteins were precipitated by addition of 0.6 g of ammonium sulfate per milliliter of extract. The extract was centrifuged at 10,000 × *g* for 30 min at 4°C, and the supernatant was removed. The pellet (precipitated proteins) was solubilized in 300 μl of 100 mM phosphate buffer containing 2 mM β-mercaptoethanol and 10% glycerol, and stored at −80°C.

### Detection of Antioxidant Enzyme Activities

Ascorbate peroxidase activity was detected as described in Ratkevicius et al. (2003). To determine AP activity, 50 μg was added to 1 ml of reaction solution containing 100 mM phosphate buffer (pH = 7) and 16 mM hydrogen peroxide. The decrease in absorbance due to ascorbate consumption was detected at 290 nm for 1 min.

Dehydroascorbate reductase (DHAR) activity was determined as described in Ratkevicius et al. (2003). To determine DHAR activity, 70 μg of protein extract was added to 1 ml of reaction mixture containing 100 mM phosphate buffer (pH = 7), 1 mM GSH, and 0.5 mM dehydroascorbate (DHA). The increase in absorbance at 290 nm due to ASC synthesis was determined at 290 nm for 1 min.

Glutathione reductase activity was detected as described in Ratkevicius et al. (2003). To determine GR activity, 50 μg of protein extract was added to 1 ml of reaction mixture containing 100 mM phosphate buffer (pH = 7), 0.5 mM of oxidized glutathione (GSSG), and 0.15 mM NADPH. The decrease in absorbance due to NADPH consumption was detected at 340 nm for 1 min.

### Preparation of Nonprotein Thiol Extracts

The alga (0.2 g of DT) was frozen in liquid nitrogen and homogenized in a mortar with a pestle and extracted by addition of 1.2 ml of 0.1% (w/v) trifluoroacetic acid (TFA)–6.3 mM diethylenetriaminepentaacetic acid (DTPA). The mixture was centrifuged at 12,000 × *g* for 20 min at 4°C, and the supernatant was recovered and filtered through a 0.45-μm-pore size membrane. Thiol groups were subjected to derivatization by mixing 250 μl of the filtered homogenate with 45 μl of 200 mM HEPES, pH = 8.2–6.3 mM DTPA, and 1 μl of monobromobimane (Invitrogen, Eugene, OR, United States), and the solution was incubated at room temperature in darkness for 30 min. Derivatization process was arrested by addition of 30 μl of methanesulfonic acid (MSA).

### Detection of Glutathione and Phytochelatins

Detection of GSH and PCs was performed as described in Mellado et al. (2012). A sample of nonprotein thiols (20 μl) was separated by high-performance liquid chromatography (HPLC) in a reverse phase C-18 column at 25°C. GSH and PCs were eluted using 0.1% TFA aqueous solution (solvent A) and 100% acetonitrile (solvent B) and a linear gradient of 0 to 20% of solvent B for 10 min, 20–35% of solvent B for 30 min, and 35–100% of solvent B for 10 min, a flow rate of 1 ml min^–1^, and 200 bars of pressure. GSH and PCs were detected by fluorescence at 380-nm excitation and 470-nm emission wavelengths. Retention times of GSH, PC2, and PC4 were 9.6, 12.7, and 16.7 min, respectively. The calibration curve was prepared with 1 to 50 nmol of GSH.

### Detection of Ascorbate

Detection of ASC was performed as described in Ratkevicius et al. (2003). The alga (0.5 g of DT) was homogenized with liquid nitrogen in a mortar using a pestle. Five milliliters of 2.5 M perchloric acid was added, and the homogenization was pursued until thawing. The homogenate was centrifuged at 10,000 × *g*, and the supernatant was recovered. A sample of 150 μl was added to a mixture containing 2% (w/v) TCA, 8.8% ortho-phosphoric acid, 0.01% α,α′-dipyridyl, and 10 mM ferric acid in a final volume of 1 ml. The reaction mixture was incubated 1 h at 40°C, and the absorbance was determined at 525 nm. The calibration curve was prepared using 1–300 nmol of ASC.

### Extraction of Total RNA

The extraction of total RNA was performed as described in Navarrete et al. (2019). The alga (0.1 g of FT) was homogenized in liquid nitrogen in a mortar with a pestle. One milliliter of TRIzol reagent (Life technologies, Carlsbad, CA, United States) was added, and the homogenization was pursued until thawing. The mixture was centrifuged at 10,000 × *g* for 10 min at 4°C, and the supernatant was recovered. Chloroform (200 μl) was added, and the mixture was vortexed for 10 s and left at room temperature for 3 min. The mixture was centrifuged at 10,000 × *g* for 15 min at 4°C, and the upper aqueous phase was recovered. Isopropanol (500 μl) was added, and the solution was incubated at room temperature for 10 min; the solution centrifuged at 10,000 × *g* for 10 min at 4°C, and the supernatant was discarded. The pellet (total RNA) was washed with 1 ml of 75% ethanol and centrifuged at 10,000 × *g* for 5 min. Ethanol was removed, and the pellet was dried at room temperature for 15 min. The pellet was solubilized in 15 μl of ultrapure water treated with DEPC and incubated for 15 min with 1 U of DNAse I, and the enzyme was inactivated by heating at 65°C for 10 min. Total RNA was quantified using the kit Quant-iT Ribogreen RNA assay (Invitrogen, United States), the ratio of absorbance 260/280 nm was determined, RNA integrity was verified in an agarose gel stained with ethidium bromide, and total RNA was stored at −80°C.

### Quantification of Metallothionein Relative Transcript Levels

The relative level of transcripts encoding MTs of *U. compressa*, UcMT1, UcMT2, and UcMT3, were determined by qRT-PCR using a real-time thermocycler (Agilent, United States). The cDNA was prepared using reverse transcriptase (Bio-Rad, United States) and oligo-dT and 1 μg of total RNA. Amplifications were performed using 50 ng of total cDNA, 15 ng of primers, and 3 mM magnesium chloride. Tubulin-β was used as housekeeping gene since the levels of transcripts did not change under cadmium stress in *U. compressa* (D. Laporte, personal communication). PCR primers were designed based on previously obtained transcriptomes of *U. compressa* (Laporte et al., 2016, 2020a) and they were: TUB-F: 5′TGCAACTTTTGTAGGCAACTC3′ and TUB-R: 5′CAGTGAACTCCATCTCGTCC3′; UcMT1-F: 5′CCAGTGCC AAACCGAAGATG3′ and UcMT1-R: 5′TGCTAGCAG GCACAGTCGTC3′; UcMT2-F: 5′GCACTCCTGAGACCT GCACT3′ and UcMT3-R: 5′ATCCTTCGCGGGTGAGCAAG3′; UcMT3-F: 5′TCTTGTTGTGAAGCAAGTGA3′ and UcMT3-R: 5′CACAGTTGCATTCTGCGGTT3′. Amplification was performed for 5 s at 95°C, 10 s at 54°C for tubulin, or 10 s at 56°C for UcMTs, and 40 cycles of amplification in all cases. The relative level of transcripts was expressed as 2^–ΔΔ*CT*^ (Livak and Schmittgen, 2001).

### Statistical Analyses

Statistical analyses were performed using Statgraphics Centurion 16 software followed by two-way ANOVA Tukey’s test at a confidence interval of 95%.

## Results

### Kinetics of Intracellular Cadmium Accumulation in *Ulva compressa*

**FIGURE 1:**
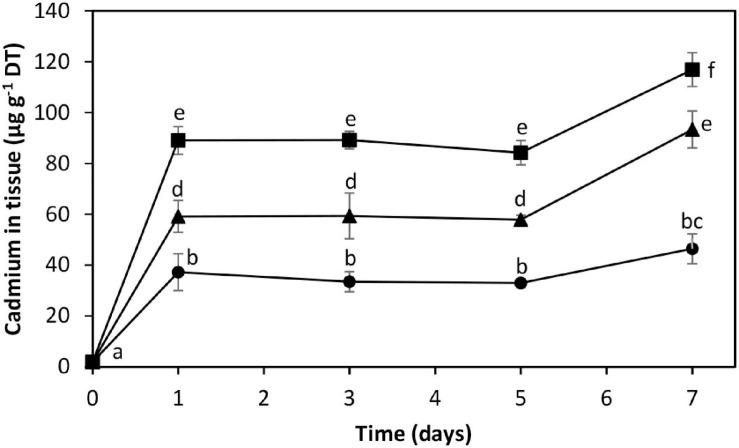
Accumulation of intracellular cadmium in the marine alga *Ulva compressa* cultivated in seawater with 10 μM (black circles), 25 μM (black triangles), and 50 μM (black squares) of cadmium for 0 to 7 days. The level of intracellular cadmium is expressed as micrograms per gram of dry tissue (DT). Symbols represent the mean values of three independent experiments. Different letters indicate significant differences among mean values (±SD) (*P* < 0.05).

The alga was cultivated in seawater with increasing concentrations of cadmium corresponding to 10, 25, and 50 μM of the metal (Figure 1). The level of intracellular cadmium was 2 μg g^–1^ of dried tissue (DT) in control condition. The alga cultivated with 10, 25, and 50 μM cadmium showed an increase in intracellular cadmium reaching 37.2, 59.2, and 89.1 μg g^–1^ of DT, respectively, on day 1, intracellular cadmium level remained unchanged until day 5, and it increased again on days 7 to 46.4, 93.4, and 116.9 μg g^–1^ of DT, respectively (Figure 1). Thus, the kinetics of intracellular cadmium accumulation showed a triphasic kinetic pattern of cadmium accumulation with an initial increase on day 1, a plateau until day 5, and a second increase on day 7.

### Oxidative Stress in Response to Cadmium Stress

**FIGURE 2:**
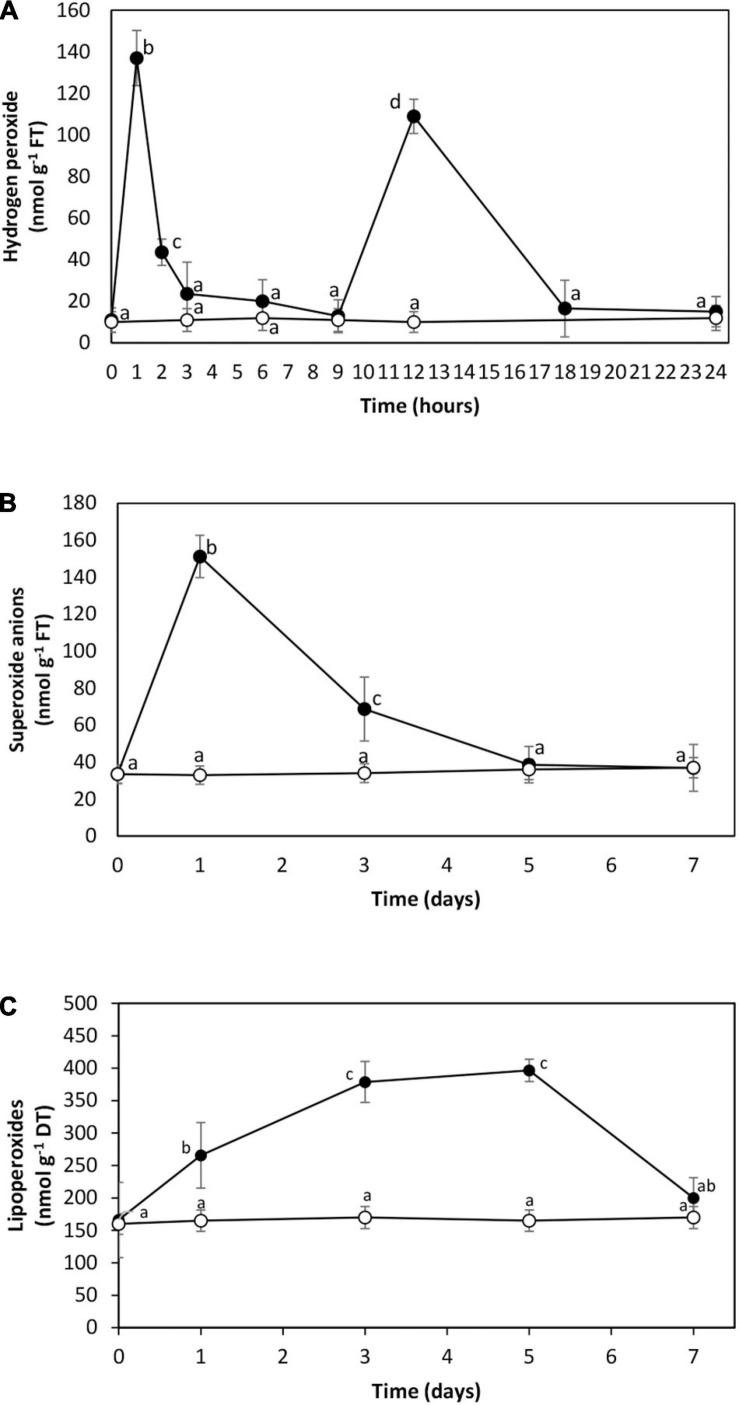
Level of hydrogen peroxide **(A)**, superoxide anions **(B)**, and lipoperoxides **(C)** in the marine alga *U. compressa* cultivated in seawater without copper addition (open circles) and with 10 μM of cadmium (black circles) for 0 to 7 days. The level of hydrogen peroxide is expressed as nanomoles per gram of fresh tissue (FT), that of superoxide anions as nanomoles per gram of FT, and that of lipoperoxides as nanomoles per gram of dry tissue (DT). Symbols represent mean values of three independent experiments. Different letters indicate significant differences among mean values (±SD) (*P* < 0.05).

The level of hydrogen peroxide was 11 nmol g^–1^ of fresh tissue (FT) in control condition, and it increased to 137 nmol g^–1^ of FT at 1 h of cadmium exposure, then it decreased to 20 nmol g^–1^ of FT at 3 and 6 h, to 13 nmol g^–1^ of FT at 9 h of exposure, increased again to 109 nmol g^–1^ of FT at 12 h of exposure and decreased to 15 nmol g^–1^ of FT until 24 h of exposure (Figure 2A). The level of hydrogen peroxide level did not increase at 48 or 72 h of cadmium exposure (data not shown). The level of superoxide anions was 33 nmol g^–1^ of FT in control condition. It increased to 151 nmol g^–1^ of FT on day 1 and decreased to reach the control level on days 5 and 7 (Figure 2B). The level of lipoperoxides was 166 nmol g^–1^ of DT in control condition. It increased to 397 nmol g^–1^ of DT on day 5 and decreased to 200 nmol g^–1^ of DT on day 7 (Figure 2C). Thus, cadmium excess induced oxidative stress reflected by increases in hydrogen peroxide at 2 and 12 h, superoxide anions with a maximal level on day 1, and lipoperoxides with a maximal level on days 3 to 5 in *U. compressa*.

### Activation of Antioxidant Enzymes in Response to Cadmium Stress

**FIGURE 3:**
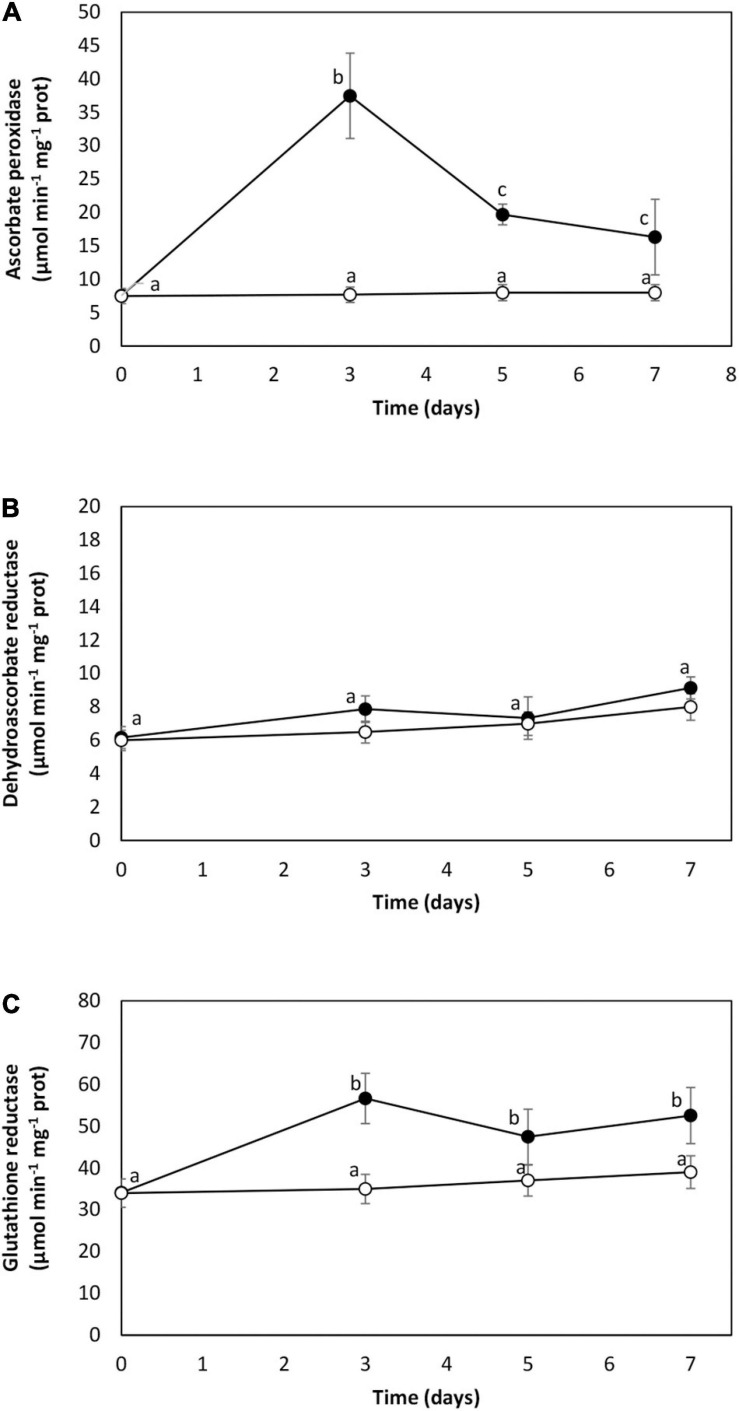
Activities of antioxidant enzymes ascorbate peroxidase **(A)**, dehydroascorbate reductase **(B)**, and glutathione reductase **(C)** in the marine alga *U. compressa* cultivated in seawater without cadmium addition (open circles) and with 10 μM of cadmium (black circles) for 0 to 7 days. Enzyme activities are expressed as micromoles per minute per gram of proteins. Symbols represent mean values of three independent experiments. Different letters indicate significant differences among mean values (±SD) (*P* < 0.05).

The activity of AP was 8 μmol min^–1^ mg^–1^ of protein in the control condition. It increased to 38 μmol min^–1^ mg^–1^ of protein on day 1 and decreased to 16 μmol min^–1^ mg^–1^ of protein on day 7 (Figure 3A). The activity of DHAR was 7 μmol min^–1^ mg^–1^ of protein in the control and remained unchanged until day 7 of cadmium exposure (Figure 3B). The activity of GR was 34 μmol min^–1^ mg^–1^ of protein in the control, and it increased to 56 μmol min^–1^ mg^–1^ of protein on day 3 and remained increased until day 7 (Figure 3C). Thus, cadmium excess activates antioxidant enzyme system in *U. compressa*.

### Synthesis of Glutathione and Ascorbate in Response to Cadmium Stress

**FIGURE 4:**
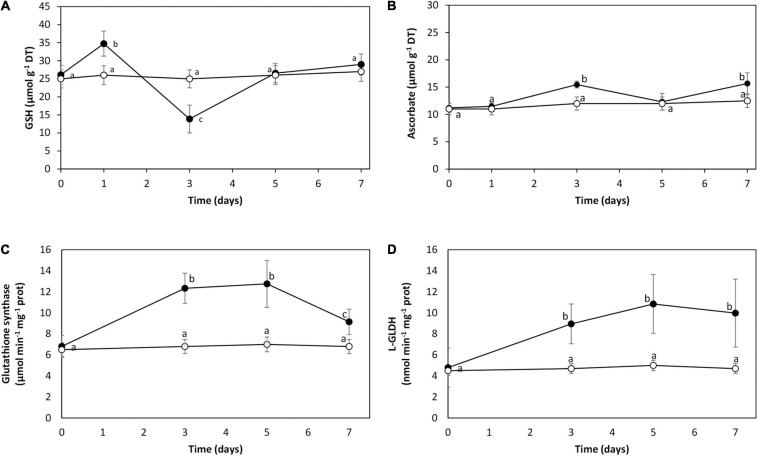
Level of reduced glutathione (GSH, **A**) and ascorbate (ASC, **B**) and activities of enzymes glutathione synthase (GS, **C**) and L-galactono lactone dehydrogenase (L-GLDH, **D**) in the marine alga *U. compressa* cultivated in seawater without cadmium addition (open circles) and with 10 μM of cadmium (black circles) for 0 to 7 days. The level of GSH is expressed in micromoles per gram of dry tissue (DT) and ascorbate in micromoles per gram of fresh tissue (FT). Symbols represent mean values of three independent experiments. Different letters indicate significant differences among mean values (±SD) (*P* < 0.05).

The level of GSH was 26 μmol g^–1^ of DT in control condition. It increased to 35 μmol g^–1^ of DT on day 1, decreased until 14 μmol g^–1^ of DT on day 3, and increased to reach the control level on days 5 and 7 (Figure 4A). The level of ASC was 11 μmol g^–1^ of FT in control condition, and it slightly increased on days 5 and 7 to 15 μmol g^–1^ of DT (Figure 4B). The activity of the enzyme glutathione synthase (GS) involved in GSH synthesis was 6.8 μmol min^–1^ mg^–1^ of protein in control condition. It increased to 12 μmol min^–1^ mg^–1^ of protein on days 3 and 5, and decreased to 9 μmol min^–1^ mg^–1^ of protein on day 7 (Figure 4C). The activity of L-galactono 1,4 lactone dehydrogenase (L-GLDH) involved in the synthesis of ASC was 5 μmol min^–1^ mg^–1^ of protein in control condition, and it increased to 10 μmol min^–1^ mg^–1^ of protein on days 3, 5, and 7 (Figure 4D). Thus, cadmium excess induced the synthesis of GSH and ASC in *U. compressa*, mainly GSH.

### Synthesis of PCs and Increase in Expression of UcMTs in Response to Cadmium Stress

**FIGURE 5:**
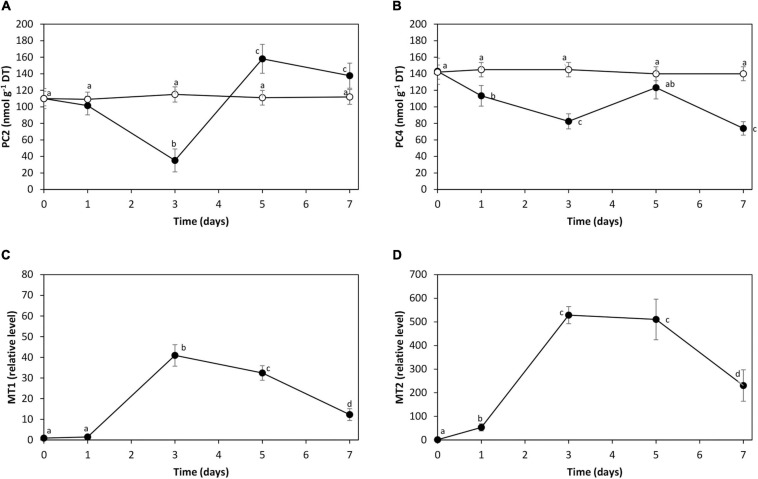
Level of phytochelatins PC2 **(A)** and PC4 **(B)** in the alga *U. compressa* cultivated in seawater without cadmium addition (open circles) and with 10 μM of cadmium (black circles) for 0 to 7 days. Relative level of transcripts encoding UcMT1 **(C)** and UcMT2 **(D)** in the alga cultivated with 10 μM of cadmium for 0 to 7 days. The levels of PC2 and PC4 are expressed as nanomoles per gram of dry tissue (DT), and the relative levels of transcripts encoding UcMT1 and UcMT2 are expressed as 2^– ΔΔ*CT*^. Symbols represent mean values of three independent experiments. Different letters indicate significant differences among mean values (±SD) (*P* < 0.05).

The level of PC2 was 110 nmol g^–1^ of DT in control condition. It decreased to 35 nmol g^–1^ of DT on day 3 and increased to 140 nmol g^–1^ of DT on days 5 and 7 (Figure 5A). The level of PC4 was 143 nmol g^–1^ of DT in control condition. It decreased to 83 nmol g^–1^ of DT on day 3, increased to 123 nmol g^–1^ of DT on day 5, and decreased to 74 nmol g^–1^ of DT on day 7 (Figure 5B).

The relative level of transcripts encoding UcMT1 showed an increase of 41 times on day 3 and 12 times on day 7 (Figure 5C). The relative level of UcMT2 increased 528 times on day 3 and 230 times on day 7 (Figure 5D). Cadmium excess did not induce the increase in the relative level of UcMT3 (data not shown). Thus, cadmium excess induced the synthesis of PC2 and PC4 and the increase in expression of UcMT1 and UcMT2 in *U. compressa*.

### Involvement of Calcium-Dependent Protein Kinases, Calmodulin-Dependent Protein Kinases, Calcineurin B-Like Protein Kinases, and MAPKs in Cadmium Accumulation, in Glutathione and Phytochelatin Synthesis and in the Expression of Metallothioneins

**FIGURE 6:**
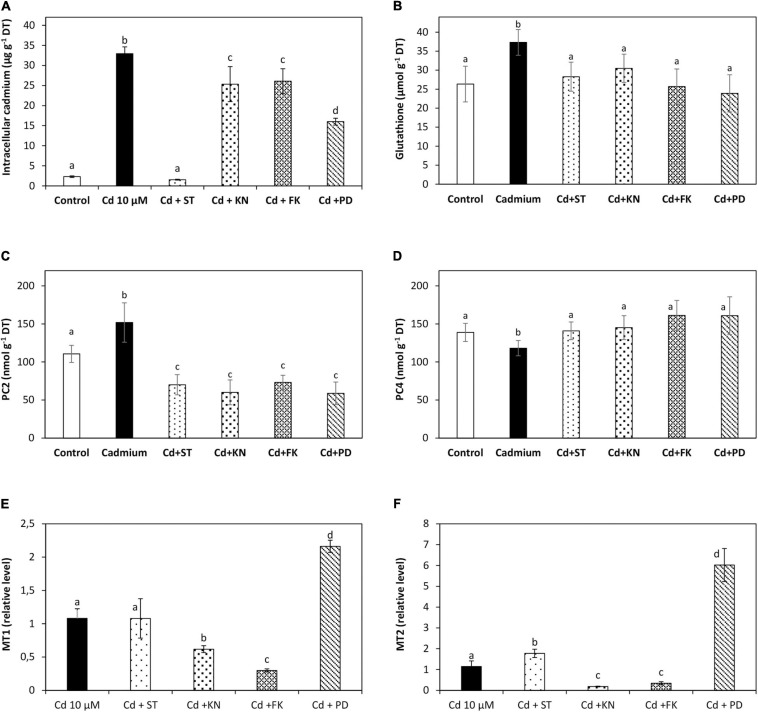
Level of intracellular cadmium **(A)**, glutathione (GSH, **B**), phytochelatins PC2 **(C)**, and PC4 **(D)** and transcripts of the metallothioneins UcMT1 **(E)** and UcMT2 **(F)** in the alga *U. compressa* cultivated in seawater without cadmium addition (control), with 10 μM of cadmium, and 5 μM of inhibitors staurosporine (ST), KN62 (KN), with FK506 (FK) and PD98059, PD, and with 10 μM of cadmium for 5 days. The level of intracellular cadmium is expressed as micrograms per gram of dry tissue (DT), GSH, and PCs are expressed as nanomoles per gram of DT, and the level of transcripts encoding UcMT1 and UcMT2 are expressed as 2^– ΔΔ*CT*^. Bars represent mean values of three independent experiments. Different letters indicate significant differences among mean values (±SD) (*P* < 0.05).

The level of intracellular cadmium was 2 μg g^–1^ of DT in control condition, and it was 33 μg g^–1^ of DT in the alga cultivated with 10 μM of cadmium for 5 days. Staurosporine (ST), an inhibitor of CDPKs, decreased the level of intracellular cadmium to 1.5 μg g^–1^ of DT on day 5; KN62 (KN), an inhibitor of CaMKs, decreased the level of intracellular calcium to 25 μg g^–1^ of DT; FK506 (FK), an inhibitor of CBLPKs, decreased the level of intracellular cadmium to 26 μg g^–1^ of DT; and PD98059 (PD), an inhibitor of MAPKs, decreased the level of intracellular cadmium to 16 μg g^–1^ of DT (Figure 6A). Thus, CDPK, CaMK, CBLPK, and MAPK signaling pathways are involved in cadmium accumulation in *U. compressa*.

The level of GSH was 37.3 μmol g^–1^ of DT in the alga cultivated with 10 μM cadmium for 5 days (Figure 6B). ST decreased the level of GSH to 28.3 μmol g^–1^ of DT; KN decreased its level to 30.5 μmol g^–1^ of DT; FK decreased its level to 25.7 μmol g^–1^ of DT; and PD decreased its level to 23.9 on day 5 (Figure 6B). The level of PC2 was 3.4 nmol g^–1^ of DT in the alga cultivated with 10 μM of cadmium for 5 days (Figure 6C). ST decreased the level of PC2 to 1.6 nmol g^–1^ of DT; KN decreased its level to 1.3 nmol g^–1^ of DT; FK decreased its level to 1.6 nmol g^–1^ of DT; and PD decreased its level to 1.3, on day 5 (Figure 6C). The level of PC4 decreased on day 5 to 188 nmol g^–1^ of DT but increased the level on day 5 (Figure 6D). Thus, CDPK, CaMK, CBLPK, and MAPK signaling pathways are involved in GSH and PCs synthesis in *U. compressa* exposed to cadmium stress.

The relative level of transcripts encoding UcMT1 remained unchanged in the alga treated with ST and 10 μM of cadmium; it decreased in the alga treated with KN and FK; and it increased two times in the alga treated with PD (Figure 6E). Thus, CaMKs and CBLPKs are involved in the activation of UcMT1 expression, whereas MAPKs are involved in inhibition of UcMT1 expression. The relative level of transcripts of UcMT2 increased 1.5 times in the alga treated with ST and 10 μM of cadmium; it decreased in the alga treated with KN and FK; and it increased 5.2 times in the alga treated with PD (Figure 6F). Thus, CaMKs and CBLPKs are involved in the activation of UcMT2 expression, CDPKs and MAPKs are involved in the inhibition of UcMT2 expression, and UcMTs could be involved in cadmium accumulation, but their involvement may be less relevant than the increase in GSH and PCs levels.

## Discussion

### Kinetics of Intracellular Cadmium Accumulation in *Ulva compressa*

In this work, we showed that increasing concentrations of cadmium ranging from 10 to 50 μM induced a triphasic kinetic pattern of intracellular cadmium accumulation in the marine alga *U. compressa*. This first step was an increase in intracellular cadmium on day 1, then a plateau until day 5, and a second increase in intracellular cadmium on day 7. This triphasic kinetic contrast with the linear accumulation of cadmium in *U. lactuca* cultivated with 0.4 to 0.7 μM cadmium for 4 days (Kumar et al., 2010), and with the linear accumulation observed in *U. prolifera* and *U. linza* cultivated with 0 to 80 μM cadmium for 7 days (Jiang et al., 2013). Thus, the kinetics of cadmium accumulation differs among Ulvophyceae.

The plateau of cadmium accumulation observed from days 1 to 5 may be due to cadmium extrusion to the extracellular medium, as it has been previously observed in the alga cultivated with copper ions (Navarrete et al., 2019). In fact, *U. compressa* released copper ions to the culture medium accompanied by an equimolar amount of GSH, but not PCs or MTs (Navarrete et al., 2019). The release of the cadmium to the culture medium could be accompanied in this case by GSH and/or PCs since both decreased on day 3. In plants, it has been shown that ABCC1 and ABCC2 transporters also participate in the transport of cadmium/PC complexes to the vacuole that are further released to the culture medium (Park et al., 2012). In this sense, it has been shown in *E. gracilis* cultivated with cadmium that the metal accumulates in the chloroplast and showed an increased protein level of transporter HMT2 that extrudes cadmium to the extracellular medium and HMT3 that allows its accumulation in the chloroplasts (Kathiwada et al., 2020). Thus, transporters similar to the HMT family may exist in *U. compressa*, as in green microalgae and euglenoids (Hainikenne et al., 2005; Kathiwada et al., 2020). The second increase in intracellular cadmium may be mediated by GSH and PCs, mainly PC2, since the inhibition of their synthesis using inhibitors of signal transduction pathways correlate with the decrease in intracellular cadmium.

### Cadmium-Induced Oxidative Stress and Activation of Antioxidant Enzymes

Cadmium excess induced the accumulation of hydrogen peroxide at 2 and 12 h, the increase of superoxide anions on day 1 that decreased until day 5, and the increase in lipoperoxides on days 1 to 5 that decreased to control level on day 7. Thus, cadmium induced an oxidative stress condition in *U. compressa* reflected by the increase in superoxide anions and hydrogen peroxide levels and the production of lipoperoxides. In this sense, it has been shown that *U. compressa* cultivated with 10 μM of copper showed an increase in hydrogen peroxide at 2, 3, and 12 h, a continuous increase in superoxide anions from day 3 until day 7 (González et al., 2010), and an increase in lipoperoxides began on day 3 reaching a plateau on days 5 to 7 (A. Moenne, unpublished data). The higher increase in superoxide anions induced by copper stress may be due the higher electronegativity of copper, which is 1.95 and that of cadmium, which is 1.65, and the latter may lead to a higher capture of electrons in electron transport chains in the mitochondria and chloroplasts (González et al., 2010). Thus, cadmium and copper induced an oxidative stress condition in *U. compressa*, as it has been shown in plants (Cuypers et al., 2011, 2016; Romero-Puertas et al., 2019).

The buffering of superoxide anions is due to SOD that produces hydrogen peroxide, and hydrogen peroxide is reduced by an antioxidant, the enzyme AP, to oxygen and water. In plants, AP is coupled to DHAR and GR constituting the Halliwell–Asada–Foyer cycle (Foyer and Noctor, 2011). Considering that the superoxide anion level decreased on day 7, the activity of SOD might be increased, and hydrogen peroxide produced by SOD was mitigated by the activation of AP that was not coupled to GR since DHAR activity was not increased. However, GR was increased suggesting that GR may be used to recover GSH from GSSG using the reducing power of NADPH. It has been previously shown that cadmium induced oxidative stress and activation of antioxidant enzymes in different plants and microalgae (Cuypers et al., 2011, 2016; Figueira et al., 2014; Kovacik and Dresler, 2018). Thus, the activation of antioxidant enzymes that mitigate oxidative stress induced by cadmium is a common feature to all photosynthetic organisms.

### Cadmium-Induced Synthesis of Glutathione and Ascorbate

Cadmium induced the synthesis and consumption of GSH and ASC, but the production and consumption of GSH was higher than the production and consumption of ASC. The higher consumption of GSH was observed on day 3 as well as the higher consumption of PC2 and PC4, and thus, GSH may be used for the synthesis of PC2 and PC4 in order to chelate cadmium ions. As mentioned before, it is possible that GSH and/or PCs may be used to extrude cadmium ions from the cells of *U. compressa*. Results obtained with cadmium contrast with those observed in the alga cultivated with 10 μM of copper showing that ASC is constantly consumed from days 1 to 7, and the activity of AP strongly increased from days 3 to 7 (González et al., 2010; Mellado et al., 2012). Thus, cadmium tolerance involved mainly GSH synthesis, whereas copper tolerance is based mostly on ASC synthesis in *U. compressa*.

### Cadmium-Induced Synthesis of Phytochelatins and Increased Expression of Metallothionein

Cadmium accumulation was observed on day 1, and synthesis and consumption of PCs began on day 1. In contrast, the expression of UcMTs occurred on days 3 to 5, and the second increase in intracellular cadmium was observed on day 7. Thus, it is possible that the initial accumulation of cadmium was mediated by GSH and PCs, and the second phase of accumulation may involve PCs and/or UcMTs. In this sense, it has been shown that PCs mediate cadmium accumulation in plant vacuoles through transporters HMT3 that transport free cadmium ions, HMT1 that transport cadmium with GSH and ABCC1, and ABCC2 that transport cadmium with PCs (Mendoza-Cózatl et al., 2011; Shukla et al., 2012, 2013) as well as MTs (Zimeri et al., 2005; Pagani et al., 2012; Rono et al., 2021). In contrast, *U. compressa* cultivated with 10 μM copper showed an increase in PC2 and PC4 on days 5 to 12, and no decrease in their levels compared with cadmium, and this increase was concomitant with enhanced expression of UcMT occurring on days 5 to 12 (Navarrete et al., 2019). In addition, particles containing copper are accumulated in the chloroplast of *U. compressa* (D. Espinoza, unpublished), and probably, cadmium may also be accumulated in chloroplasts, as it has been shown in *E. gracilis* treated with cadmium (Mendoza-Cózatl et al., 2002; Mendoza-Cózatl and Moreno-Sánchez, 2005). Thus, PCs and MTs are involved in copper and cadmium accumulation in *U. compressa*, probably in chloroplasts.

Interestingly, cadmium increased the expression of UcMT1 and UcMT2, but not UcMT3. The latter contrasts with the results obtained in the alga cultivated with copper that showed the increase in the expression of the three UcMTs (Navarrete et al., 2019). In addition, cadmium mainly increased the expression of UcMT2, compared with UcMT1, since these increases were 41 and 528 times, respectively. This contrasts with results obtained in the alga cultivated with copper that showed that increases in UcMT1, UcMT2, and UcMT3 were similar, and the level of transcripts increased only 10–15 times. Thus, the effects of cadmium stress are quite different from those of copper stress in *U. compressa*, and cadmium induced a higher consumption of PCs and a higher expression of UcMT1 and UcMT2.

### Involvement of Calcium-Dependent Protein Kinases, Calmodulin-Dependent Protein Kinases, Calcineurin B-Like Protein Kinases, and MAPKs in Cadmium Accumulation, in the Increase in Glutathione and Phytochelatin Levels and Overexpression of Metallothionein

Regarding the involvement of CDPKs, CaMKs, CBLPKs, and MAPK signaling pathways in cadmium accumulation, and GSH and PC synthesis and UcMTs expression, it was shown that cadmium accumulation required the activation of the four signaling pathways since their inhibition leads to a decrease in cadmium accumulation, mainly CDPKs. In addition, the level of GSH decreased, the level of PC2 decreased, and the level of PC4 increased on day 5 with the four inhibitors. These results strongly suggest that GSH and PCs may be involved in cadmium accumulation in *U. compressa*. In addition, the expression of UcMT1 and UcMT2 decreased with KN and FK indicating that CaMKs and CBLPKs are involved in the increased expression of MTs induced by cadmium. Even if UcMT expression is inhibited only by two inhibitors, it is not excluded that UcMTS may also be involved in cadmium accumulation in *U. compressa*. In this sense, it has been shown that cadmium is sequestered in the vacuoles of plants and yeast (Speiser et al., 1992; Li et al., 1997) or in the chloroplast in *E. gracilis* through the binding to PCs and/or GSH (Mendoza-Cózatl et al., 2002; Mendoza-Cózatl and Moreno-Sánchez, 2005). In tomato cells resistant to cadmium, in plants such as *Brassica juncea* and *Silene vulgaris*, and in the fission yeast *Schizosaccharomyces pombe*, cadmium and copper were accumulated as a complex with PCs and sulfide, and this complex is more stable and showed a higher affinity for these metals than PCs (Murasugi et al., 1983; Steffens et al., 1986; Reese and Winge, 1988; Reese et al., 1988; Verkleij et al., 1990; Speiser et al., 1992). Recently, it has been determined that copper accumulates in the chloroplasts of *U. compressa* as copper-containing electrodense nanoparticles as visualized by transmission electron microscopy (TEM) coupled with energy dispersive X-Ray spectroscopy (EDXS) (D. Espinoza, unpublished). Thus, it is possible that cadmium and copper may accumulate in the chloroplast of *U. compressa* bound to GSH, PC, sulfide, and/or UcMT, but the latter need to be further determined.

## Conclusion

**FIGURE 7:**
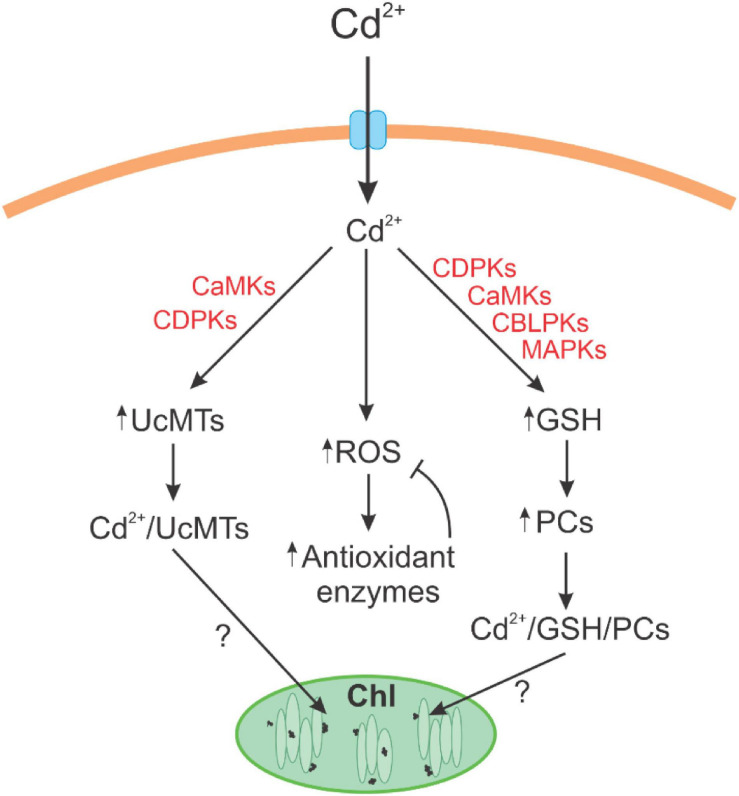
Scheme of cadmium tolerance and accumulation in the marine alga *Ulva compressa*. Cadmium induced the synthesis of reactive oxygen species (ROS) that are buffered by antioxidant enzymes; cadmium induced the synthesis of glutathione (GSH) and phytochelatins and the increase in metallothionein (UcMT) expression. The increase in cadmium accumulation and in GSH and PC levels involved the activation of CDPKs, CaMKs, CBLPKs, and MAPKs signaling pathways, and the increase in UcMTs expression involved only CaMKs and CBLPKs. GSH, PCs, and/or UcMTs may participate in the formation of electrodense nanoparticles in the chloroplast of *U. compressa* allowing cadmium accumulation.

The alga *U. compressa* cultivated with cadmium showed the accumulation of the metal at intracellular level showing a triphasic kinetic pattern. Cadmium induced increases in hydrogen peroxide and mainly of the level of superoxide anions leading to the production of lipoperoxides indicating that cadmium induced an oxidative stress condition in the alga. Oxidative stress was mitigated by activation of antioxidant enzymes and the synthesis of GSH and ASC, mainly GSH. Cadmium induced the synthesis and consumption of PCs suggesting that PCs are involved in cadmium extrusion and/or accumulation in the alga. Cadmium also induced an increased expression of UcMTs. The inhibition of signaling pathways dependent on CDPKs, CaMKs, CBLPKs, and MAPKs leads to the inhibition of cadmium accumulation and to a decreased level of GSH and PC2 and an increase in PC4. In contrast, only inhibitors CaMK and CBLPKs decreased the level of UcMTs expression (see scheme in Figure 7). Thus, it is possible that GSH and PCs are involved in cadmium accumulation in chloroplasts of *U. compressa*, but it is not excluded that UcMTs may also participate in its accumulation.

## Data Availability Statement

Publicly available datasets were analyzed in this study. This data can be found here: https://doi.org/10.6084/m9.figshare.14039546.

## Author Contributions

AG and DL did experimental work. AM designed the experiments and wrote the manuscript. All authors contributed to the article and approved the submitted version.

## Conflict of Interest

The authors declare that the research was conducted in the absence of any commercial or financial relationships that could be construed as a potential conflict of interest.
